# Breathe Easy EDA: A MATLAB toolbox for psychophysiology data management, cleaning, and analysis

**DOI:** 10.12688/f1000research.13849.2

**Published:** 2018-12-14

**Authors:** John C. Ksander, Sarah M. Kark, Christopher R. Madan

**Affiliations:** 1 Department Psychology, Boston College, Chestnut Hill, MA, USA; 2 Department Psychology, Brandeis University, Waltham, MA, USA; 3 School of Psychology, University of Nottingham, Nottingham, UK

**Keywords:** respiration artifact, electrodermal activity, skin conductance

## Abstract

Electrodermal activity (EDA) recordings are widely used in experimental psychology to measure skin conductance responses (SCRs) that reflect sympathetic nervous system arousal. However, irregular respiration patterns and deep breaths can cause EDA fluctuations that are difficult to distinguish from genuine arousal-related SCRs, presenting a methodological challenge that increases the likelihood of false positives in SCR analyses. Thus, it is crucial to identify respiration-related artifacts in EDA data. Here we developed a novel and freely distributed MATLAB toolbox, Breathe Easy EDA (BEEDA). BEEDA is a flexible toolbox that facilitates EDA visual inspection, allowing users to identify and eliminate respiration artifacts. BEEDA further includes functionality for EDA data analyses (measuring tonic and phasic EDA components) and reliability analyses for artifact identification. The toolbox is suitable for any experiment recording both EDA and respiration data, and flexibly adjusts to experiment-specific parameters (e.g., trial structure and analysis parameters).

## Introduction

Electrodermal activity (EDA) methods evaluate fluctuations in skin electrical conductance caused by changes in sweat gland production. The sympathetic nervous system innervates palmar and plantar eccrine sweat glands, and changes in skin conductance are thought to measure sympathetic nervous system arousal ( Bach *et al.*, 2010). Importantly, EDA recordings are a valuable and popular psychophysiological measurement in studies of affect and cognition ( Boucsein *et al.*, 2012).

It is well known that respiration and EDA influence each other ( Schneider *et al.*, 2003). In laboratory settings, researchers often leverage this relationship to check the integrity of a psychophysiology set-up. Asking participants to take a deep breath should produce concurrent deflections in both waveforms, and properly configured recording equipment should detect that response. EDA is typically recorded using electrodes placed on the palmar or plantar surfaces where eccrine sweat glands are densely located. Respiration, typically recorded using a belt secured around the diaphragm, is an oscillatory event that approximates a sine wave with regular breathing. However, irregular respiration, or abnormalities in the respiration waveform (frequency or amplitude), are associated with non-specific changes in the EDA waveform. These physiological respiration-related artifacts can lead researchers to overestimate the presence or magnitude of skin conductance responses (SCRs) in experiments ( Schneider *et al.*, 2003).

Despite the strong relationship observed between EDA and respiration traces, prior work has shown that EDA and respiratory signals are not *strictly* coupled ( Rittweger *et al.*, 1996), which may relate to differences in their physiological origin. Physiologically, the emotion-reactive palmar and plantar eccrine sweat glands are maximally innervated by cholinergic ( Sato & Sato, 1981) sudomotor fibers leaving the ventral root of the spinal cord ( Boucsein, 2012, p. 20). While eccrine sweat glands are modulated by the sympathetic nervous system, the transmission related to EDA is mainly cholinergic, not noradrenalergic ( Sato & Sato, 1981; Stern *et al.*, 2001). However, deep breathing has been associated with sudden increases in free-circulating adrenaline, producing sweat responses ( Boucsein, 2012, p. 32), which mimic SCRs on EDA recordings. As mentioned above, this relationship is useful for checking psychophysiological signal integrity, but can also bias SCR analyses.

While movement-induced EDA artifacts are fairly straightforward to identify (e.g., presence of an unusually steep rise in the waveform), physiologically derived artifacts appear similar to arousal-related waveforms ( Boucsein, 2012). Developing methods for identifying respiration-related artifacts has been a challenge for the field of psychophysiological research due to high intersubject and intrasubject variability in respiration activity, yielding a wide range of waveform characteristics ( Schneider *et al.*, 2003). A lack of analytical solutions has motivated software development within this field since the early 1990’s, with the goal of improving how researchers inspect and manipulate respiration data ( Wilhelm & Roth, 1993).

Researchers are strongly encouraged to account for such respiration-induced EDA artifacts, and subsequently outline those artifact elimination procedures in their manuscripts ( Boucsein *et al.*, 2012). This can be challenging, since common artifact-control practices involve researchers visually inspecting their respiration data, which is unfortunately both time-consuming and subjective. Schneider *et al.* (2003) has provided a useful decision tree for discarding artifact EDA responses based on a set of criteria. However, an easy-to-use and freely available software that expedites visual inspection of respiration data, and allows researchers to quantify their artifact-control procedures is not available. This toolbox might be particularly helpful for researchers identifying respiration artifacts in experiments with longer trial durations, such as viewing video clips or recalling autobiographical memories. In these experiments, the standard stimulus-response latency window for identifying event-related SCRs (e.g., 1–4 seconds) may no longer be suitable, and longer trials almost certainly have a higher probability of respiration-related SCR artifact contamination.

Currently, there is a need for easy-to-use, flexible, and interoperable software that facilitates EDA artifact elimination via the widely employed and accepted method of visual inspection. We have developed a novel MATLAB toolbox for efficiently eliminating EDA respiration artifacts and analyzing EDA data, which we freely distribute as Breathe Easy EDA or ‘BEEDA’. BEEDA’s streamlined artifact removal interface allows users to quickly identify and clean EDA data, expediting EDA analysis without compromising analysis integrity. Additionally, BEEDA’s integrated EDA analysis functionality allows users to seamlessly analyze cleaned EDA data within the toolbox. Furthermore, the toolbox includes inter-rater reliability (IRR) analyses so that researchers may evaluate the reliability of their artifact-control procedures.

**Figure 1.**
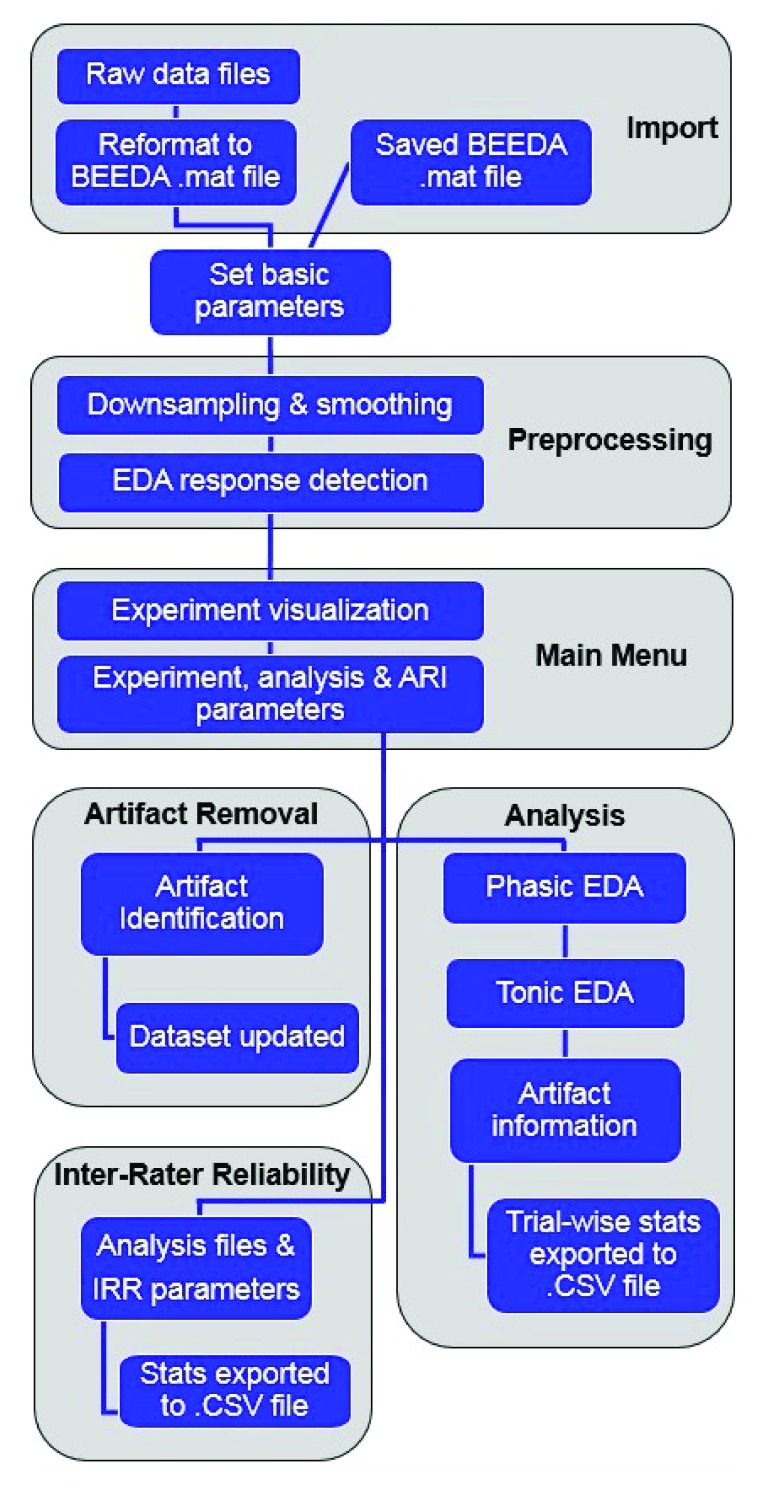
Illustration of the Breathe Easy EDA (BEEDA) workflow.

The BEEDA toolbox is controllable through a graphical user interface (GUI), and requires no programming skill to use. This toolbox may be used either for simple artifact detection, EDA analyses, or for both artifact elimination and subsequent EDA analyses—as illustrated in Figure 1. This flexibility allows users to take advantage of BEEDA’s functionality without restricting the use of complementary software such as Mindware (MindWare Technologies Ltd., Gahanna, OH), Ledalab ( Benedek & Kaernbach, 2010), ANSLAB ( Wilhelm & Peyk, 2005), or AcqKnowledge ( Braithwaite *et al.*, 2013). For instance, one could use BEEDA only for marking artifacts in a dataset, and then use the artifact information file BEEDA produces with an alternative EDA analysis program. Furthermore, BEEDA is suitable for any experiment where both EDA and respiration data were collected, and parameters specific to individual experiments can easily be modified through the GUI (e.g., trial structure and analysis options). This permits a great deal of functional flexibility, without encumbering the toolbox’s usability. Here we describe the toolbox design, workflow, and functionality.

## Toolbox design and workflow

### Overview

**Figure 2.**
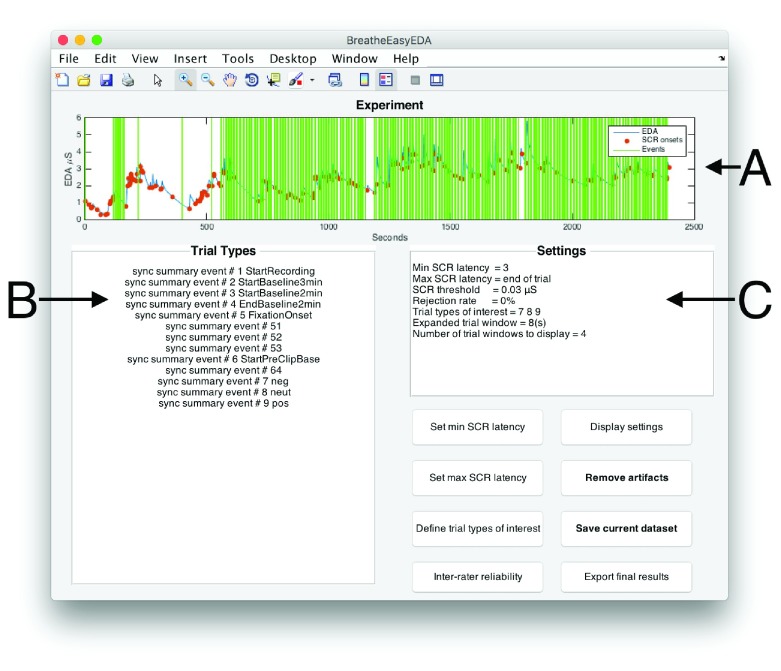
Main menu after data has been loaded into BEEDA. (A) Visual experiment summary; the EDA timecourse is plotted in blue, red points mark valid SCR onsets, and vertical green lines mark recording events (e.g. trial onsets). (B) Trial-type window displays each type of recording event imported with the dataset. (C) Current settings.

BEEDA’s workflow was designed to offer users situationally-specific functionality within the simplest framework possible. This allows researchers to use the toolbox for their specific goals, without the toolbox adding unnecessary work in the process. As illustrated in Figure 1, the BEEDA workflow begins with loading a dataset and setting a few critical parameters. After that initialization, the GUI main menu ( Figure 2) lets researchers tailor their own workflow to their specific needs. This workflow is flexible to include any combination of data visualization, artifact inspection/cleaning, calculating EDA statistics, or performing interrater reliability (IRR) analyses. The degree of overhead imposed by the workflow (e.g., in specifying parameters or manipulating the data) at this stage should only match the requirements of the user. The following sections describe these abilities and their implementation in detail.

### A. Loading an experiment into BEEDA

Initializing the BEEDA toolbox (executing *BreatheEasyEDA.m*) immediately launches the data loading GUI. This interface allows users to either load data files for a new session, or load data from a previously saved session. If a new session is started, BEEDA copies and reformats raw data files into a MATLAB structure variable ( *BEEDAdata*). The *BEEDAdata* variable is the toolbox’s primary data structure; all user defined parameters (e.g. analysis settings) and analysis actions (e.g. artifact removal) are written to this *BEEDAdata* structure. Resuming a previous session reads information from a saved *BEEDAdata* structure and launches into the main menu.

For new sessions, basic analysis parameters are also specified in the data loading GUI. These basic settings are: downsampling and Skin Conductance Response (SCR) parameters. Importantly, once downsampling and SCR options are chosen, these settings are permanently fixed for the current BEEDA session (even if the session is saved and resumed). If a downsampling factor is specified, both the EDA and respiration data are immediately downsampled within *BEEDAdata*. This downsampling functionality is provided because the sampling rate capabilities of modern EDA systems (e.g. >1000 Hz) far exceed the resolution necessary for EDA analyses. Downsampling datasets to lower temporal resolutions can dramatically reduce a dataset’s size, consequently improving BEEDA’s memory and hard disk requirements, computation time, and GUI responsiveness.

### B. Main menu

 The main menu provides a visual summary of your experiment, trial information, analysis settings, and display settings ( Figure 2A). The main menu also allows users to save the current BEEDA session, start the artifact removal interface, run IRR analyses, and export final analysis results.

 Before displaying the experiment summary panel, the EDA data is first smoothed via convolution with a Gaussian kernel (as in Benedek & Kaernbach (2010)). Smoothing removes minor signal noise, which may originate from a variety of sources (e.g. recording equipment or downsampling). Next, valid SCRs are identified based on previously specified threshold and rejection-rate parameters. The experiment summary panel plots the entire experiment’s EDA timecourse, marking onset times for trials, and valid SCRs ( Figure 2A). This window provides users with an overview of the experiment’s EDA data, allowing users to easily confirm the indented dataset has loaded correctly.

 All unique trial-types are displayed in the trial-type information window, and the current BEEDA session’s settings are displayed in the setting information window ( Figure 2B and 2C). From the main menu, users can easily set a number of session settings: SCR latency tolerances, valid trials for analysis, and display settings (see ** Interface display options). SCR latency tolerances establish the stimulus time-locked window when SCRs may be appropriately attributed to the preceding stimulus (see Main EDA analysis parameters), typically a 3-second window between 1–4 seconds post-stimulus onset ( Boucsein, 2012), but shorter windows have been proposed (e.g., 2 seconds or less; Barry, 1990; Levinson & Edelberg, 1985). Additionally, if end-of-trial events were omitted during an experiment’s data collection, specifying a maximum SCR latency parameter effectively creates these events. Specifying the valid trials for analysis determines which trial-types are available for artifact cleaning and EDA analysis. All unique events recorded during data collection may be declared as valid trial-types; this allows users to disregard inter-trial events, baseline events, or events not corresponding to trials of interest.

### C. Interface display options

The “Display settings” ** main menu button ( Figure 2) allows users to customize the Artifact Removal Interface. The *Expanded trial window* parameter controls the additional timecourse data displayed before and after each trial in the artifact removal interface. For instance, setting expanded trial window to 5 (seconds) will display the 5 seconds before every trial and the 5 seconds after every trial. This option may help users evaluate how respiration immediately preceding or following a trial relates to respiration during a trial. More specifically, we found that being presented with the activity surrounding the trial provided a useful context for identifying potential respiration artifacts.

 The *Number of trial windows to display* parameter controls the number of trials simultaneously displayed in the artifact removal interface. This option may be particularly useful when running the BEEDA toolbox on computers with lower resolution computer monitors, as users can adjust the number of trials in each ARI page to best fit their display configuration.

### D. Artifact removal interface

**Figure 3.**
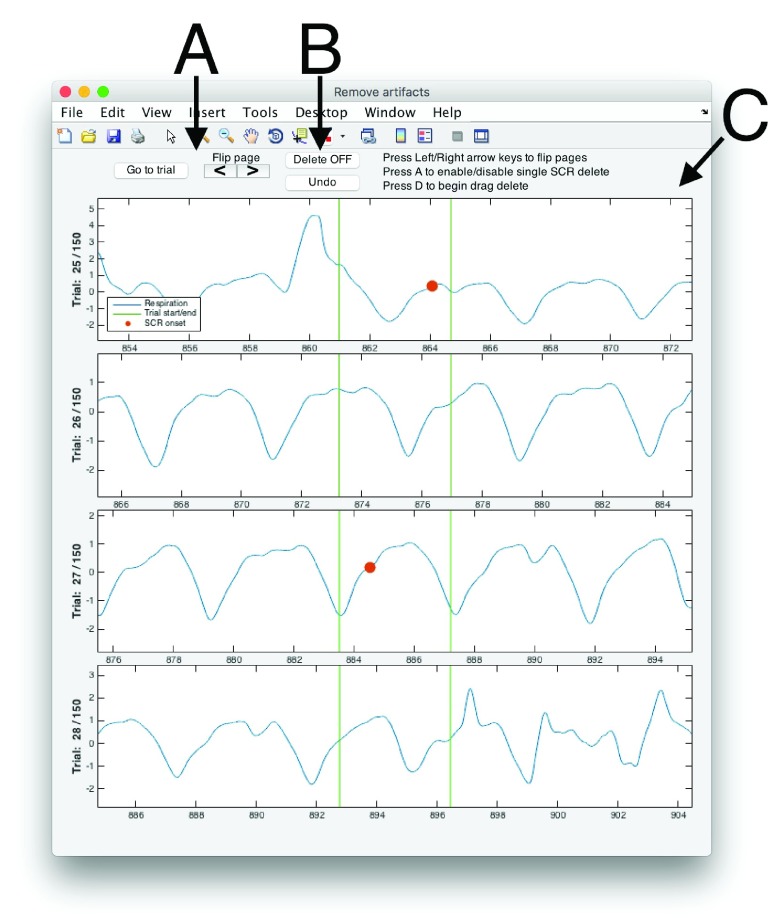
Artifact removal interface displays a page of four trials. (A) Event navigation controls. (B) Data manipulation controls and hotkey guide. (C) Respiration timecourse is plotted in blue, red points mark valid SCR onsets, and vertical green lines mark an event’s start and end.

Selecting “Remove artifacts” from the main menu will launch the Artifact Removal Interface (ARI). The ARI allows users to efficiently clean EDA data via streamlined data presentation and easy to use controls. Users can easily scroll through ‘pages’ of trials, examining each trial for irregular respiration waves, as shown in Figure 3. If problematic respiration waves are identified, users can clean the data with either ‘SCR delete mode’ or ‘drag-delete mode’. Drag-delete mode removes entire time segments of EDA data, whereas SCR delete mode only removes SCRs from analysis consideration. Consequently, drag delete mode is recommended for Skin Conductance Level (SCL) analyses and thorough artifact elimination, whereas SCR delete mode is only recommended for SCR analyses (see EDA analysis functionality). 

**Figure 4.**
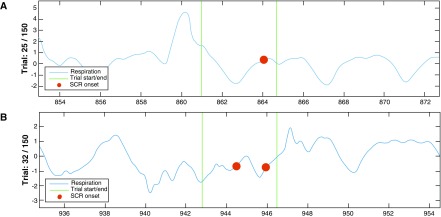
Two examples of artifact SCRs displayed in the artifact removal interface. The presentation simplifies inspecting data for a sudden deep breath (Panel **A**) or highly irregular breathing pattern (Panel **B**) preceding an SCR onset.

In the ARI, user defined trials of interest are individually displayed by plotting SCR onset timepoints directly onto the trial’s respiration data ( Figure 3C). This presentation simplifies the manual identification of problematic breathing (e.g. Figure 4), and the recommended procedures for EDA respiration artifact scrubbing can be found in Schneider *et al.* (2003). All user actions (e.g., data cleaning) are immediately applied to *BEEDAdata* and can be saved through the main menu.

### E. Exporting results and artifact information

Selecting “Export final results” in the main menu will analyze the user-defined trials-of-interest and export the analysis results to a Comma Separated Values formatted spreadsheet (.CSV file). This spreadsheet will show trial-wise EDA statistics, in addition to whether or not the trial was flagged for artifacts. A trial will show “flagged for artifacts” if any SCR or data segment was deleted from the trial. In this way, one may simply use BEEDA’s GUI to mark artifacts within an EDA dataset, then use the artifact information output with another EDA analysis software. Similarly, the artifact information output provides an easy means for assessing overall data quality. Experimenters may also directly analyze this output with BEEDA, in order to evaluate how reliably artifacts were identified within a dataset.

## Artifact inter-rater reliability

To facilitate the reporting and validity of respiration artifact rejection ** methods *,* BEEDA includes inter-rater reliability (IRR) analysis functionality for respiration artifact rejection. Selecting “Inter-rater reliability” in the main menu will perform an artifact IRR analysis directly on exported BEEDA result files. This requires that researchers have cleaned a dataset multiple times, under the same relevant parameters (verified by built-in sanity checks). After specifying these files, users can set the IRR analysis’ scope to match their analysis goals. Specifically, users can limit their analysis to only trials containing SCRs (as defined by SCR threshold parameters) or analyze all trials of interest. This is a critical distinction, as the IRR for SCR-negative trials may give unrepresentative reliability statistics for SCR oriented analyses (i.e. trials without SCRs may not have been inspected). On the other hand, these trials would certainly be considered for SCL analyses. This choice determines *T* in the subsequent equations.

 After setting the IRR scope, the pair-wise Cohen’s κ between all raters is calculated and exported to a CSV spreadsheet as a labeled matrix. We used Cohen’s κ implementation ( Cohen, 1960) in this context:

$$\kappa =\frac{p_{o}-p_{e}}{1-p_{e}}$$

For the set of all trials *T* we defined two trial classes *C* as: the absence of any artifact marking, or the presence of any SCR/data-segment deletion. The expected chance agreement, *p _e_*, between each pair of raters *i* and *j* was:

$$p_{e}=\frac{1}{|T{|}^{2}}\sum_{k=1}^{2}|T_{i}^{C_{k}}||T_{j}^{C_{k}}|$$

where $\left|T_{i}^{C_{k}}\right|$ would be the number of trials in class *C _k_* for rater *i*. The observed rater agreement, *p*_*o*_, was:

$$p_{o}=\frac{1}{\left|T\right|}\sum_{k=1}^{2}|T_{i}^{C_{k}}\cap T_{j}^{C_{k}}|$$

The user-guide documentation describes how this analysis and its output (i.e. the labeled Cohen’s κ matrix) are configured in greater detail.

## EDA analysis functionality

 The BEEDA toolbox features integrated EDA analysis functionality, which may be used with or without prior artifact removal. Selecting the *Export final results* main menu button will initialize EDA analyses and export the subsequent results as a spreadsheet. These analyses measure tonic and phasic EDA using standard methodology ( Boucsein, 2012). Tonic EDA is defined as the slow change in SCLs over a timecourse of interest. BEEDA determines the mean and standard deviation of each trial’s EDA levels, and these statistics are included in the results output. Data segments marked as artifacts using the *Drag delete mode* are not included in SCL analyses.

Phasic EDA measurements are determined via the trough-to-peak detection of SCRs ( Boucsein, 2012). SCRs are quickly changing EDA levels that exceed an amplitude threshold and occur within a response window time-locked to a stimulus. The SCR amplitude is defined as the SCR’s peak EDA level minus the SCR’s initial trough EDA level. Users can explicitly specify an SCR amplitude threshold, and this practice is typical for trough-to-peak SCR detection. Alternately, the amplitude threshold can be flexible and data driven via setting an SCR rejection rate ( Kim *et al.*, 2004). In BEEDA, specifying an explicit SCR threshold of 0μS and a rejection rate of 10% emulates the algorithmic SCR thresholding procedure described in Kim *et al.* (2004). While this thresholding procedure is not typically employed, BEEDA includes this functionality to mirror proprietary EDA analysis software packages which offer similar analysis options ( Braithwaite *et al.*, 2013).

For phasic EDA analyses, BEEDA detects valid SCRs and exports the following statistics for each trial: number of SCRs, average SCR magnitude, cumulative SCR magnitude, and maximum SCR magnitude. SCRs in data segments removed with *Drag delete mode,* in addition to SCRs marked as artifacts with *SCR delete mode*, are not included in SCR analyses.

BEEDA’s does not include functionality for hypothesis testing with EDA statistics. Instead, the analysis results are written to a long-format .CSV spreadsheet with comprehensive labeling. The common data and file formatting allow users to easily run their hypothesis testing with any commonly used software package (e.g. SPSS, R, etc), without arduous file-conversions or reformatting.

## Implementation details for signal processing and analyses

The imported raw data is first smoothed according to the following procedure (as also implemented in Benedek & Kaernbach (2010)). The EDA signal is iteratively smoothed with a Guassian kernel, increasing the standard deviation on each iteration until there is negligible reduction in the signals’ root mean square of successive differences (RMSSD), or until a maximum standard deviation of 125 ms. More explicitly, for an EDA recording *X* with *t *timepoints sampled at *f Hz*, and a Gaussian kernel *G* specified with *μ* = 0 and *σ* = .125 *h*, the algorithm follows this pseudocode:

Initialize *h* = 0
Initialize RMSSD as *ε*_*old*_ = $\sqrt{\frac{1}{N}\sum_{t=2}^{N}{\left(X_{t}–X_{t-1}\right)}^{2}}$
Initialize *ε*_*th*_ = 10 ^–5^, *ε*_*new*_ = 0, and Δ *ε* = *ε*_*old*_
While *ε*_*th*_ > Δ *ε* and *f* > *h* :
*h* = *h* + 4
*σ* = .125 *h*
  The Gaussian kernel is specified with this new *σ*
$G=\frac{1}{\sigma \sqrt{2\pi }}e^{\frac{-{\left(x-\mu \right)}^{2}}{2\sigma^{2}}}$
*X* = *X* * *G*
*ε*_*new*_ = $\sqrt{\frac{1}{N}\sum_{t=2}^{N}{\left(X_{t}–X_{t-1}\right)}^{2}}$
  Δ *ε* = *ε*_*new*_ – *ε*_*old*_
*ε*_*old*_ = *ε*_*new*_

Following this initial smoothing, any requested downsampling is performed via decimation. The data is then resmoothed with the previously described algorithm, and this concludes the smoothing procedures.

BEEDA implements trough-to-peak SCR detection for the EDA recording *X* with the first derivative $\frac{dx}{dt}$ for each timepoint (Δ *X*_*t*_). SCR trough indices *O* are defined by a positive rate following negative rates:

$$O=\{t|\Delta X_{t-1}+\Delta X_{t}>\Delta X_{t-1}\}$$

SCR peak indices *P* are defined by a negative rate following positive rates:

$$P=\{t|\Delta X_{t-1}-\Delta X_{t}>\Delta X_{t-1}\}$$

This implementation was constrained such that the first trough index must precede the first peak index, and the last trough index must precede the last peak index (i.e. elements of *O* and *P* must form trough-to-peak pairings). The SCR amplitudes were then simply calculated as *R*_*i*_ = *X*_*P _i_*_ – *X*_*O _i_*_. In the following section describing BEEDA’s EDA analysis statistics, the equations will follow the notation in this section. Additionally, *T* will describe the set of all experimental trials, with specific trails indexed as *T*_*j*_. The set of responses belonging to a given trial will be indexed as $R_{i}^{T_{j}}$, and likewise a set of timepoints in given trial will be indexed as $X_{t}^{T_{j}}$. 

## EDA analysis statistics

- *Number of SCRs*: the number of valid SCRs in a trial: *n*( $R_{i}^{T_{j}}$)
- *Average SCR magnitude*: average trial SCR amplitude: $\frac{1}{n(R_{i}^{T_{j}})}\sum_{i=1}^{n}R_{i}^{T_{j}}$
- *Max SCR magnitude*: the largest SCR amplitude within a trial: max( $R_{i}^{T_{j}}$)
- *Cumulative SCR magnitude*: the sum of all trial SCR amplitudes: $\sum_{i=1}^{n}R_{i}^{T_{j}}$
- *SCL(average)*: mean EDA signal within a trial: $\frac{1}{n(X_{t}^{T_{j}})}\sum_{t=1}^{n}X_{t}^{T_{j}}$
- *SCL(standard deviation)*: the standard deviation of a trial’s EDA signal: $\sqrt{Var(X_{t}^{T_{j}})}$

## Main EDA analysis parameters

- *SCR threshold*: Only EDA responses above this amplitude threshold are considered valid SCRs. Typically an amplitude threshold of .05μS is used, although some researchers advocate for thresholds as low as .01μS ( Braithwaite *et al.*, 2013). Schmidt & Walach (2000) recommend that sampling resolution should be taken into account when considering low thresholds, and thresholds lower than .01μS should not be used.
- *Rejection rate*: If a rejection rate greater than 0 is specified, trial-wise thresholding is applied according to: *R _th_* = max⁡( *R*) *α* where *R _th_* is the trial-specific response threshold, *R* is the trial’s set of responses and *α* is the rejection rate. For example, if the rejection rate is 10% and a trial’s largest SCR amplitude is 4μS, SCRs with amplitudes below .4 μS are rejected in that trial.
- *Min SCR latency*: The minimum time after a trial’s start when EDA data can be considered for analyses (i.e. the stimulus response window). Valid SCRs onsets must begin after the specified minimum latency time, and EDA levels before minimum latency time will be excluded from SCL analyses. Benedek & Kaernbach (2010) report that a minimum latency of 1 second post-stimulus is typical.
- *Max SCR latency*: The time after a trial’s start when EDA data cannot be considered for analyses. Valid SCRs must begin before the specified maximum latency, and EDA signal after the maximum latency is excluded from SCL analyses. Benedek & Kaernbach (2010) report that a maximum latency of 3 or 5 seconds post-stimulus is typical.

## Operation

BEEDA’s system requirements are: Matlab R2014b or newer, and the Matlab Signal Processing Toolbox. Any computer with that prerequisite Matlab software can run BEEDA (e.g. regardless of operating system). However, users are recommended to run BEEDA with Matlab R2015a, since the toolbox was developed and extensively tested with R2015a. 

 BEEDA was designed for input datasets containing both EDA and respiration recordings. However, suitable input files may also contain placeholder values for either data channel (i.e. for datasets without either respiration or EDA recordings). The toolbox was designed to accept raw data files from Biopac (Biopac Systems Inc., USA) recording systems. The BEEDA user-guide describes how these files are obtained from Biopac systems, and how these files are formatted. Although BEEDA was designed to easily accept files from these widely-used systems, any comparably formatted files are also suitable (i.e. from other recording systems). The acceptable formatting is very basic, and therefore recordings from other systems should not present major issues.

## Use case

We have provided a sample dataset ^1^. This data was collected during an emotional-image viewing experiment, and is provided for toolbox demonstration purposes. Documentation for this sample dataset is included with the distribution, and provides further background about the experiment and the data’s structure. We have also provided example analysis output using this dataset as Supplementary File 1. This output shows artifact information from data cleaning, along with the analyses described in the sections on EDA analysis functionality and statistics. The output file is formatted as a .CSV spreadsheet, with easily interpretable column headers.

## Conclusion

Breathe Easy EDA is a novel MATLAB toolbox developed for easy and reliable identification of respiration-related artifacts in EDA data. This software was specifically built to facilitate the methodical considerations of psychophysiology researchers through a simple, flexible, interoperable, and tolerant design. BEEDA’s simplified data presentation allows efficient data inspection and cleaning, without sacrificing functionality in the GUI. In fact, the intuitive interface includes features that are absent from widely used contemporary EDA software, but still essential to researchers (e.g., an “undo” function). The artifact cleaning functionality extends to integrated reliability analyses, providing a simplified means for researchers to establish the consistency of their artifact-control procedures across independent raters. BEEDA’s common output-file format and range of analysis capabilities also allows users to integrate this toolbox in their analysis pipelines without precluding alternate software packages. Furthermore, BEEDA was built to flexibility handle any experiment where both respiration and EDA data were collected, regardless of trial duration or experimental design. In these ways, this software provides researchers with optimized tools for psychophysiology analysis. The toolbox is freely available from http://github.com/johnksander/BreatheEasyEDA, and the user-guide documentation for BEEDA is included with this distribution.

## Software availability

Software/source code available from: http://github.com/johnksander/BreatheEasyEDA

Archived source code as at time of publication: https://doi.org/10.5281/zenodo.1168739 ( Ksander, 2018).

License: GNU General Public

## Notes

^1^ Figure 2– Figure 4 were produced with this data.

## Supplementary material

**Supplementary File 1:** Example output file.
