# Supplementary material for: Breathe Easy EDA: A MATLAB toolbox for psychophysiology data management, cleaning, and analysis
Source: F1000Res. 2018 Dec 14;7:216. Originally published 2018 Feb 22. [Version 2] doi: 10.12688/f1000research.13849.2 (PMC6317497; doi:10.12688/f1000research.13849.2)
Supplement: Peer review history

# Peer review: Referee response for version 2

**DOI:** 10.5256/f1000research.19055.r41970

**Published:** 2018-12-27

**Authors:** Edwin S. Dalmaijer

**Affiliations:** 1 MRC Cognition and Brain Sciences Unit, University of Cambridge, Cambridge, UK

The authors have done an excellent job incorporating my minor comments in their revision.

I have read this submission. I believe that I have an appropriate level of expertise to confirm that it is of an acceptable scientific standard.

# Peer review: Referee response for version 2

**DOI:** 10.5256/f1000research.19055.r41971

**Published:** 2018-12-17

**Authors:** Jens Foell

**Affiliations:** 1 Department of Psychology, Florida State University, Tallahassee, FL, USA

I have no further comments.

I have read this submission. I believe that I have an appropriate level of expertise to confirm that it is of an acceptable scientific standard.

# Peer review: Referee response for version 1

**DOI:** 10.5256/f1000research.15053.r33784

**Published:** 2018-05-11

**Authors:** Jens Foell

**Affiliations:** 1 Department of Psychology, Florida State University, Tallahassee, FL, USA

Review of manuscript “Breathe Easy EDA: A MATLAB toolbox for psychophysiology data management, cleaning, and analysis”

 The presented manuscript describes a newly developed Matlab toolbox named BEEDA, which was mainly designed to improve the way that researchers deal with artifacts due to respiration when working with EDA data. This is relevant as EDA is a widely used physiological measure, often in conjunction with other methods such as neuroimaging or pain stimuli, and because respiration has been shown to alter EDA-derived data in a way that can lead to false interpretations.

 The authors describe the relationship between respiratory artifacts and genuine EDA signal, including the fact the two have a non-linear interrelationship and that due to their nature, respiratory artifacts are harder to identify than, for example, artifacts caused by movement. They then describe the procedure and workflow of the toolbox that they have developed to address respiration-related issues with the analysis of EDA signals.

 Examples are given for how the toolbox will display respiration that is unusual in its frequency or intensity (irregular breathing and sudden deep breath, respectively) and how the system allows to judge the temporal instance of these events in comparison to EDA responses of interest. The user can then run trial-by-trial analysis and flag or delete suspicious events.

 The described toolbox was clearly designed to integrate all necessary parts of an EDA analysis into one package: apart from artifact identification and correction, it includes all standard EDA analysis procedures, so that the normal user should be able to rely on this toolbox alone. Further, it includes algorithms to calculate inter-rater reliability.

 While these additions are not strictly required for artifact cleaning software (as there are other software packages available that can be used to perform these tasks), I would expect them to increase convenience for the user, as this integrated system reduces the clutter caused by combining different toolboxes to work on the same data set. Further, the fact that the software package is free and includes a graphical user interface will facilitate its use by researchers within the scientific community.

 In summary, the described toolbox provides a convenient way to run a data quality assessment and subsequent data analysis for EDA studies, with a special focus on correcting for respiratory effects.

 The manuscript is written clearly and succinctly and provides all necessary information for a user to understand and use the toolbox. After a thorough review of the text, I have no recommended edits.

I have read this submission. I believe that I have an appropriate level of expertise to confirm that it is of an acceptable scientific standard.

# Peer review: Referee response for version 1

**DOI:** 10.5256/f1000research.15053.r32730

**Published:** 2018-04-05

**Authors:** Edwin S. Dalmaijer

**Affiliations:** 1 MRC Cognition and Brain Sciences Unit, University of Cambridge, Cambridge, UK

The authors present a Matlab toolbox to assess skin-conductance measurements.Their toolbox sets itself apart from existing analysis options in particular by allowing users to filter out artifacts related to movement and (partially) unrelated physiological signals that arise through e.g. breathing. The authors outline what task-related signal researchers tend to be interested in, and how artifacts can distort these. The authors also highlight a current lack of user-friendly analysis solutions, and therefore present a toolbox that should offer Matlab users a platform to do skin conductance analyses.

 I particularly enjoyed the brief and to-the-point introduction, which manages to clearly introduce the need for the presented software without unnecessary excursions. In addition, I appreciate that the authors took the time to produce this software package and to open it up to fellow researchers. The authors would benefit from a citable publication on their efforts, as unfortunately scientific software is currently under-appreciated in reward frameworks for scientists (i.e. it's hard to cite, and direct citations to software tend to be disregarded).

 The article is well-written, but summarises the algorithms included in the toolbox rather coarsely. I think the manuscript would benefit from a higher level of detail on the statistical analysis. Specifically, I think the authors should include equations that describe how parameters are computed from the signal.

 An additional feature that would benefit the software is the option to do statistical comparisons between experimental conditions, which currently does not seem to be possible. Artifact identification and correction is

 important, but also just the first step of an analysis. The target audience for this toolbox includes, according to the manuscript, people with no programming experience. These will profit from additional analysis options

 that could be built into the GUI.

 The code is made publicly available via GitHub, which is a very suitable platform. The chosen license (GNU GPL v3) allows for any kind of re-use. Unlike some open-source software, the documentation that the authors provide with their toolbox is extensive, and should (in my opinion) suffice to help even the most inexperienced of users.

 DISCLAIMER: This reviewer is not an expert in skin conductance signals, but has published open software, including toolboxes for Python and Matlab. This means I am not in a position to sufficiently review the actual software, and will thus limit my review to the manuscript.

I have read this submission. I believe that I have an appropriate level of expertise to confirm that it is of an acceptable scientific standard, however I have significant reservations, as outlined above.
